# The Early Warning and Response System (EWARS-TDR) for dengue outbreaks: can it also be applied to chikungunya and Zika outbreak warning?

**DOI:** 10.1186/s12879-022-07197-6

**Published:** 2022-03-07

**Authors:** Rocio Cardenas, Laith Hussain-Alkhateeb, David Benitez-Valladares, Gustavo Sánchez-Tejeda, Axel Kroeger

**Affiliations:** 1 Instituto Departamental de Salud de Norte de Santander-IDS, Norte de Santander, Colombia; 2 School of Public Health and Community Medicine, Sahlgrenska Academy, Institute of Medicine, Global Health, University of Gothenburg, Gothenburg, Sweden; 3 Programa de Enfermedades Transmitidas por Vector, Centro Nacional de Programas Preventivos y Control de Enfermedades, CENAPRECE, Secretaría de Salud de México, Ciudad de México, México; 4 Special Programme for Research and Training in Tropical Diseases (TDR) at the World Health Organization in Geneva, Geneva, Switzerland; 5 Centre for Medicine and Society, Master Programme Global Urban Health, Albert-Ludwigs-University Freiburg, Freiburg, Germany

**Keywords:** Zika, Chikungunya, Dengue outbreak, Early warning, Colombia, Mexico

## Abstract

**Background:**

In the Americas, endemic countries for *Aedes*-borne diseases such as dengue, chikungunya, and Zika face great challenges particularly since the recent outbreaks of CHIKV and ZIKV, all transmitted by the same insect vectors *Aedes aegypti* and *Ae. albopictus*. The Special Program for Research and Training in Tropical Diseases (TDR-WHO) has developed together with partners an Early Warning and Response System (EWARS) for dengue outbreaks based on a variety of alarm signals with a high sensitivity and positive predictive value (PPV). The question is if this tool can also be used for the prediction of Zika and chikungunya outbreaks.

**Methodology:**

We conducted in nine districts of Mexico and one large city in Colombia a retrospective analysis of epidemiological data (for the outbreak definition) and of climate and entomological data (as potential alarm indicators) produced by the national surveillance systems for dengue, chikungunya and Zika outbreak prediction covering the following outbreak years: for dengue 2012–2016, for Zika 2015–2017, for chikungunya 2014–2016. This period was divided into a “run in period” (to establish the “historical” pattern of the disease) and an “analysis period” (to identify sensitivity and PPV of outbreak prediction).

**Results:**

In *Mexico*, the sensitivity of alarm signals for correctly predicting an outbreak was 100% for dengue, and 97% for Zika (chikungunya data could not be obtained in Mexico); the PPV was 83% for dengue and 100% for Zika. The time period between alarm and start of the outbreak (i.e. the time available for early response activities) was for Zika 4–5 weeks. In *Colombia* the sensitivity of the outbreak prediction was 92% for dengue, 93% for chikungunya and 100% for Zika; the PPV was 68% for dengue, 92% for chikungunya and 54% for Zika; the prediction distance was for dengue 3–5 weeks, for chikungunya 10–13 weeks and for Zika 6–10 weeks.

**Conclusion:**

EWARS demonstrated promising capability of timely disease outbreak prediction with an operational design likely to improve the coordination among stakeholders. However, the prediction validity varied substantially across different types of diseases and appeared less optimal in low endemic settings.

## Introduction

The arbovirus diseases dengue, Zika and chikungunya are transmitted by the same insect vector *Aedes aegypti* or *Ae. albopictu*. They present an increasing public health concern in endemic countries. 3.9 billion people living in 128 tropical or sub-tropical countries are at risk of being infected with these viruses [1–4]. Currently there is no specific pharmacological treatment nor an effective vaccine for public health use so that vector management is the only measure of prevention [5]. These diseases have a high social and economic impact, particularly when they appear as outbreaks [6]. An estimate of the economic costs of a chikungunya outbreak in Colombia 2013 showed an approximate cost of 100 million dollars to the government (equivalent to 0.04% of the national gross domestic product of 2013) [7]. In the case of Mexico, a study by Undurraga et al. estimated the annual cost of dengue in Mexico to be around US $ 170 (95% CL: 151–292) million [8]. The Zika outbreaks occurring between 2015 and 2017 caused losses of 7 to 18 billion dollars in Latin America, including both the direct and indirect costs due to microcephaly and Guillain–Barré syndrome [9]. Therefore, the need for early outbreak detection (when the outbreak has already started) or better outbreak prediction (when the outbreak has not yet started) to initiate response activities and mitigate or even suppress an outbreak.

But early detection of outbreaks poses a challenge, since no universally accepted or proven sets of early warning indicators exist [10, 11]. A systematic review showed that countries are generally lacking outbreak prediction tools that can be implemented by fairly unskilled users, and can automatically manage datasets [10, 12]. In reality, in most countries the early outbreak detection—if any—is based on the increase of case numbers above a pre-established threshold (meaning that the outbreak has started already). Once an outbreak has started and the transmission rate gains speed, it will be increasingly difficult to contain the outbreak through vector control measures leading to social disruption and economic harms affected families and the society [6]. With this in mind, the Special Program for Research and Training for Tropical Diseases (TDR) at the World Health Organization (WHO) initiated together with research institutions, national dengue control services and academia in ten endemic partner countries the development of a web-based Early Warning and Response System (EWARS) for dengue, with potential uses for other arbovirus disease outbreaks [10, 13]. After initial retrospective analyses in five countries (Brazil, Mexico, Dominican Republic, Vietnam, Malaysia) [11, 14]—typically including calibration and prediction-algorithm building using historical records of diseases and alarm indicators—EWARS was tested prospectively in Brazil, Mexico and Malaysia to evaluate its qualitative and quantitative performance and user friendliness [14].

EWARS has never been tested for other emerging *Aedes*-borne diseases, such as chikungunya and Zika. This study therefore aims to test the performance of EWARS for predicting outbreaks of the three *Aedes* borne arbovirus diseases (dengue, chikungunya and Zika) using available data of the national surveillance systems in our two target countries. The response component of EWARS will not be covered in this paper.

## Methods

### Description of study sites

In both target countries the selection of study sites was dictated by the availability of information from the National Surveillance System and National Meteorological Institutes [15, 16].

**Fig. 1:**
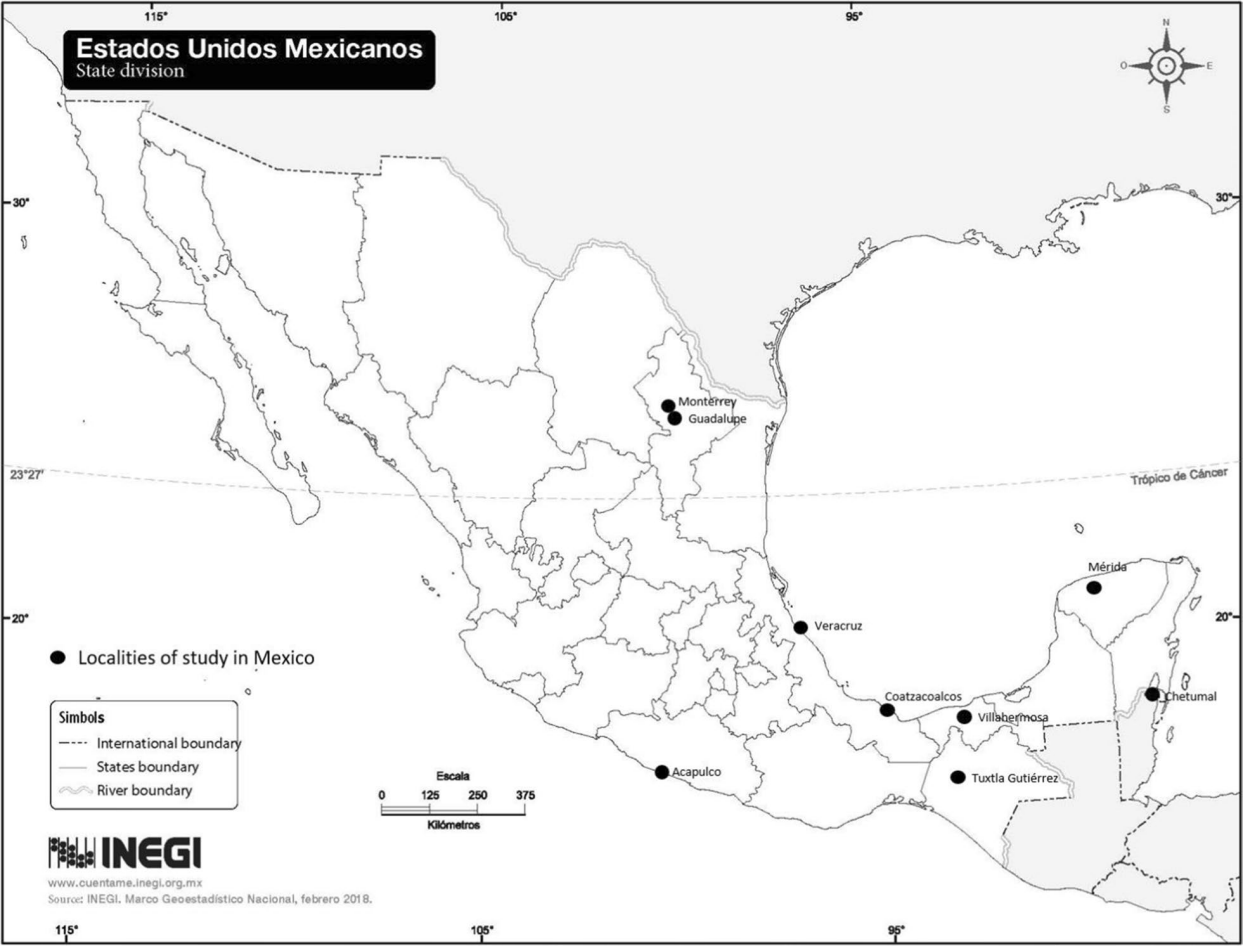
Location of study sites in Mexico

In Mexico we received the data set from nine districts (labelled as “localities”) for Zika and chikungunya as well as weekly meteorological data. In Colombia*,* we received a complete data set including weekly meteorological information and epidemiological data with individual notification for Zika and chikungunya from a city. In Mexico, from the 137 endemic urban districts (as the three disease occur mainly in urban environments due to the opportunities of vector breeding), only nine were analyzed because they had complete information records of disease and alarm indicators for the past 5 years, essential for the EWARS process. The localities selected were Tuxtla Gutierrez, Acapulco, Ciudad Guadalupe, Monterrey, Chetumal, Villahermosa, Coatzacoalcos, Veracruz and Mérida (Fig. 1). The total population of these nine districts is around 5 million inhabitants [17]. All of them possess suitable conditions for dengue vector breeding. The climate is tropical with average annual temperatures around 24 °C and rainfall above 1000 mm (except for Ciudad Guadalupe, Monterrey, and Mérida with around 700 mm).

**Fig. 2:**
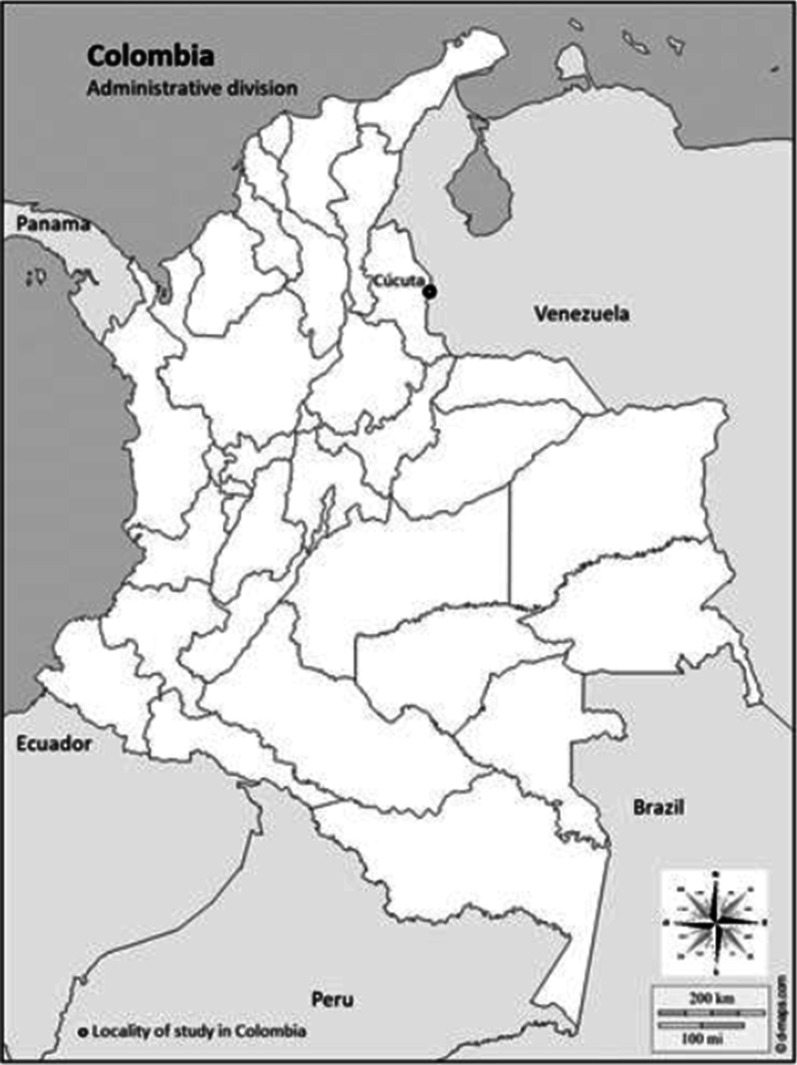
Location of study sites in Columbia

In Colombia, Cúcuta, the capital of the State (“departamento”) of Norte de Santander was included which has around 750,000 inhabitants, 1176 km^2^ in area size and is located on the border area with Venezuela (Fig. 2). The climate is tropical (temperatures ranging between 21 and 36 °C; average annual rainfall 655 mm; annual relative humidity between 70 and 75%) [18]. While dengue outbreaks have a long history in Cúcuta, outbreaks of CHIKV and ZIKV were reported only after 2014 and 2015, respectively [18].

### Public Health Surveillance system

In Mexico, the national surveillance system with its permanent notification of clinical cases of dengue, chikungunya and Zika including neurological complications feeds into the online platform SINAVENational Epidemiological Surveillance System (for “Sistema Nacional de Vigilancia Epidemiológica”) where entomological data (ovitrap indexes) and response activities (entomological platform) are registered. Both platforms receive on a weekly basis the information from all public and private health centers. Since 2018, Mexico has the fully integrated EWARS tool for dengue outbreak prediction in its national surveillance program, providing a national coverage of outbreak prediction and response activities.

In Colombia, the National Public Health Surveillance System (SIVIGILA for “Sistema Nacional de Vigilancia en Salud Pública”) provides systematic and timely information on the number of dengue, Zika and chikungunya cases. The information gathered by the centers of health care of the public and private health system, is compiled and transmitted to the SIVIGILA, which updates the results weekly [19–21]. The data for the present study corresponded to cases reported in the urban area of the city of Cúcuta. Only cases with an individual report in SIVIGILA (form 217) and with complete information required by the EWARS system were included in the analysis [22]. Data were obtained from the local surveillance system, with certified authorization. In Colombia, the EWARS for dengue is not yet been established nationally but is being tested in the city of Cúcuta, which was the actual study setting.

### Overview of the EWARS

The concept of the EWARS model is based on the Shewhart method [23] a method that adopts systematic control charts, using the historic mean and standard deviation of the outcome variable to define states of ‘in-control’ and ‘out-of-control’ throughout the model evaluation process. When applied to dengue, an Endemic Channel chart is produced which represents the number of cases within the expected normal range, or the ‘in-control’ state, while anything above this Endemic Channel (or, moving average) is considered representative of an unusual number of cases and, an ‘out-of-control’ state (i.e., an outbreak). The EWARS has advanced into an online tool (dashboard) which facilitates more structured training, applications and experience sharing among a broader range of users, also with the aid of a series of published computer-assisted user workbooks. URL: https://alramadona.shinyapps.io/Demo_Automated_Ewars/ [11, 24, 25].

**Fig. 3:**
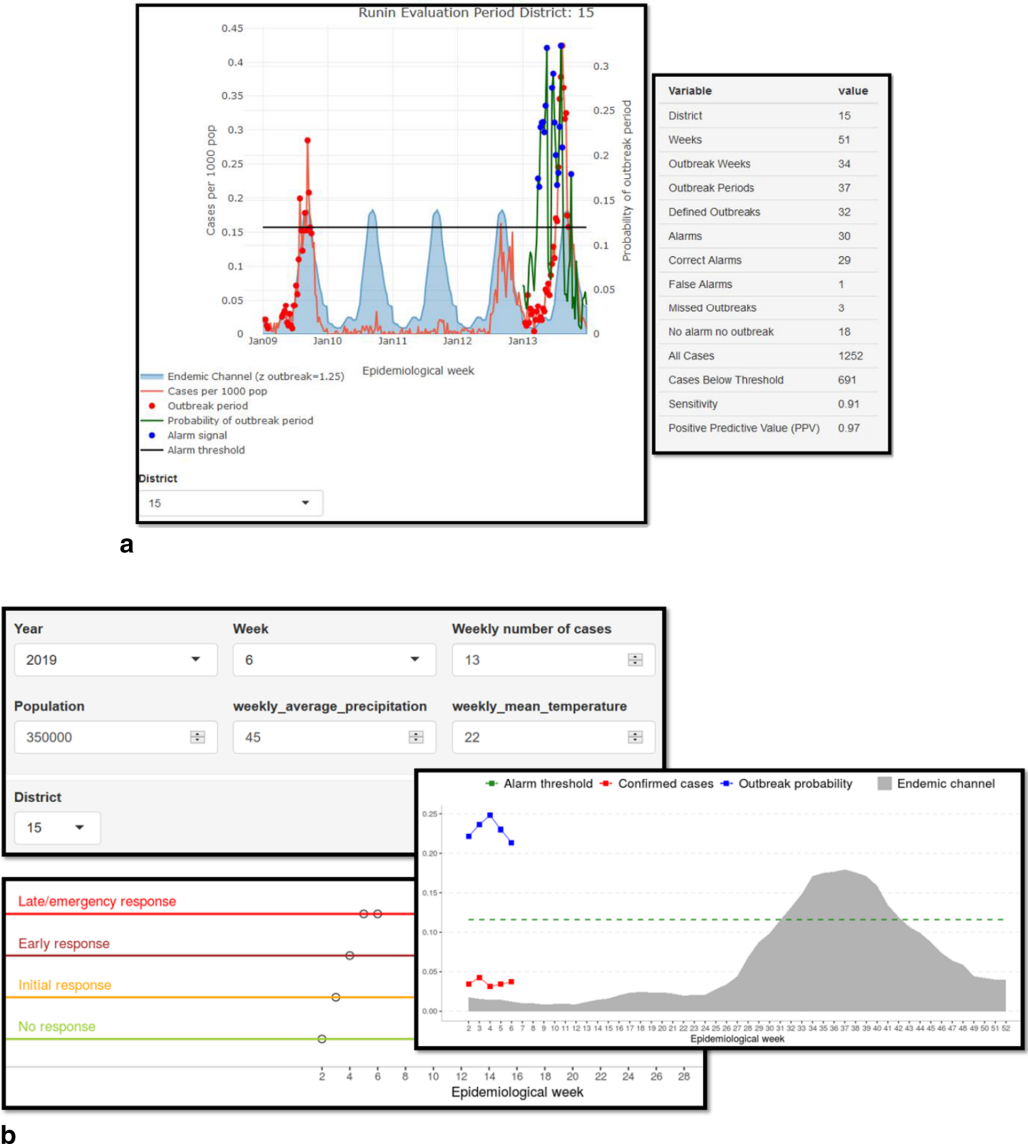
**a** Snapshot of the retrospective dashboard, including graphical and numerical visualization of the calibration process and parameters. **b** A Snapshot of theprospective dashboard, including an interface for prospective (weekly) data input prediction (alarm signal against rate of outbreaks) and structured response plan using a generic staged-response format (adoptable based on the national vector response protocol)

These user guides illustrate methodological, technical and operational aspects crucial for the tool processing. This includes details of how both digital and paper-based surveillance records of diseases and indicators can be prepared for applying in the tool. Figure 3 illustrates the elements of inputs and outputs of the EWARS tool, which is divided into retrospective (a phase of sliding window time-series cross-validation and algorithm building) and prospective (phase of inputting weekly alarm information for generating the outbreak signals) components.

### Data collection of outbreak and alarm indicators

In Mexico*,* the data collection started in January 2012 and continued until December 2018. The dataset has been provided by SINAVE and by the entomological online platform from the Secretary of Health. In Colombia, Cúcuta, the study covered the period from January 2012 to December 2017. All reported cases of dengue, chikungunya and Zika were included using the case definition for each event, according to the Ministry of Health (MoH) and National Institute of Health (NIH), set out in the public health surveillance protocols [19–21].

For the application of EWARS, the temporal unit was defined as the epidemiological ‘week’ (from Sunday to Saturday) and the spatial unit was based on pre-existing administrative units (districts). At least 3 years of surveillance data records were retained for the EWARS analysis including a variable indicating the ‘population size’ of the corresponding districts.

In the EWARS spread sheet we captured: district of residence, date of onset of symptoms, week of reporting and type of case (‘confirmed’ by laboratory or by clinical symptoms, ‘probable’ and ‘hospitalized’ cases). In addition, we also linked the potential alarm indicators such as meteorological and entomological indicators.

- *Epidemiological (disease pattern)*: weekly number of cases (confirmed cases; probable cases for chikungunya and Zika; hospitalized cases for dengue) and weekly averages of the age of the patients.
- *Meteorological (potential alarm indicators)*: mean weekly outdoor air temperature (in Celsius), relative humidity (in %), and total weekly rainfall (in mm). Data were provided by the Institute of Hydrology, Meteorology and Environmental Studies—IDEAM of Colombia; in Mexico the data were obtained from weather stations in each district [14].
- *Entomological (potential alarm indicators)*: percentage of positive ovitraps (i.e. proportion of positive ovitraps with *Aedes* eggs per week, average egg count per trap (Mexico only). (NB: Larval indices were useless as alarm indicators as they are usually not collected in a systematic way as the ovitrap system in Mexico does [10].

### Data analysis

#### Analysis by the EWARS tool

During the retrospective phase of EWARS, the weekly average number of cases for each of the three arbovirus diseases was calculated for 4 or 5 years, a year-cut off that accounted for more data variation and consequently generating higher sensitivity and PPV. These weekly averages of cases were compared with the expected “normal” or seasonal range of cases illustrated in the endemic channel [9, 10], which is the area between ± z* SD of the moving average—the z-value being the multiplier of the standard deviation of the moving average of weekly case numbers (Fig. 3). The Endemic Channel (moving average + (Z*SD)) was generated (“moving average” for each week of observation means that the average of number of cases in the 3 weeks before and after plus the week of observation were included) [11, 24, 25]. Weekly cases exceeding this Endemic Channel for two or more consecutive weeks (“outbreak window”, see below) are indicating an outbreak.

During this retrospective phase, the algorithm and all parametric coefficients needed for calculating the alarm signal are computed; these coefficients depend primarily on the sensitivity (i.e. the proportion of correctly predicted outbreaks out of all outbreaks) and positive predictive value, PPV (i.e. the proportion of correct alarms out of all alarms) as direct measures for deciding on the best calibrated settings, i.e. those with highest sensitivity and PPV. (For more details on the calibration of EWARS see the user guide by WHO-TDR) [24, 25]. The ‘alarm signal’ is triggered when the outbreak probability—which is derived from the association between alarm indicators and disease outbreak—crosses the alarm threshold (the artificial threshold that can be systematically altered to reach optimal prediction). Although the study authors have actually conducted this analysis, the algorithm calibration is usually performed by an epidemiologist at the central level (MoH). Less skilled district staff uses the prospective phase of EWARS, observing by means of the EWARS software the weekly number of cases against the upper line of the endemic channel and the pattern of alarm indicators against the pre-defined alarm threshold (Fig. 3). An alarm is triggered when the alarm indicator (‘outbreak probability’) crosses the proposed ‘alarm threshold’ [10, 14] once prospective weekly information on the relevant alarm indicator(s) are fed into the system. Accordingly, instant numerical and graphical demonstration and interpretation of a possible outbreak and its corresponding response plan is illustrated to the user at a given prediction week (time between the prediction and an outbreak to occur).

Based on recommendation from previous reports [14], ‘hospitalized’ cases proved to be the best outbreak indicator for the prediction of a forthcoming dengue outbreak using EWARS. However, when the proportion of “mild” cases is high and patients are rarely hospitalized (such as in chikungunya and Zika), ‘probable’ or ‘confirmed’ cases were used as outbreak indicators. In this study, z-values (i.e. the multiplier of SD to get the upper limit of the endemic channel) and alarm thresholds were determined for chikungunya and dengue via both manual and automated procedures (which can assess the calibration settings via a 1000 iteration process per district i.e. testing up to 1000 different cut-off values, probability thresholds and window sizes of defining outcomes and alarms) [25].

Technically, both manual and automated processing follow the same analytical process: define “window sizes” of alarm and outbreak indicators (i.e. how many weeks with an alarm indicator being above the alarm threshold to declare it an “alarm signal” and for how many weeks of case numbers being above the upper limit of the endemic channel to declare it an “outbreak”), define above which level (alarm threshold) the alarm indicator turns into an alarm signal (i.e. gives an alarm) and define the prediction distances (i.e. the time period between alarm and start of the outbreak in weeks). These variables are tested and only values that give the highest sensitivity and PPV are retained in the EWARS model. While a single alarm assessment is more straightforward, the model does take into account and adjusts for the different prediction distances associated with each alarm indicator when running multiple regressions. In this sense, the automated model is more efficient in running multiple trials and thresholds for producing the highest sensitivity and PPV for outbreak prediction.

#### Data analysis

Descriptive statistics, of both graphics and numeric formats were used in this paper. In the case of Mexico, while the prediction algorithm was run independently for each of the nine districts, output values obtained from the prediction process from each district were averaged. In Colombia, disease incidences were determined for Cúcuta (n cases / 100,000 population).

### Ethical aspects

This study analyzed only secondary data obtained from Colombian and Mexican institutions with the authorization of the Surveillance System (2017). Ethical endorsement was obtained from the Ethics Committee of the University of Freiburg (N°-145/18) which was approved by local health authorities.

## Results

### Epidemiological features of outbreaks

**Fig. 4:**
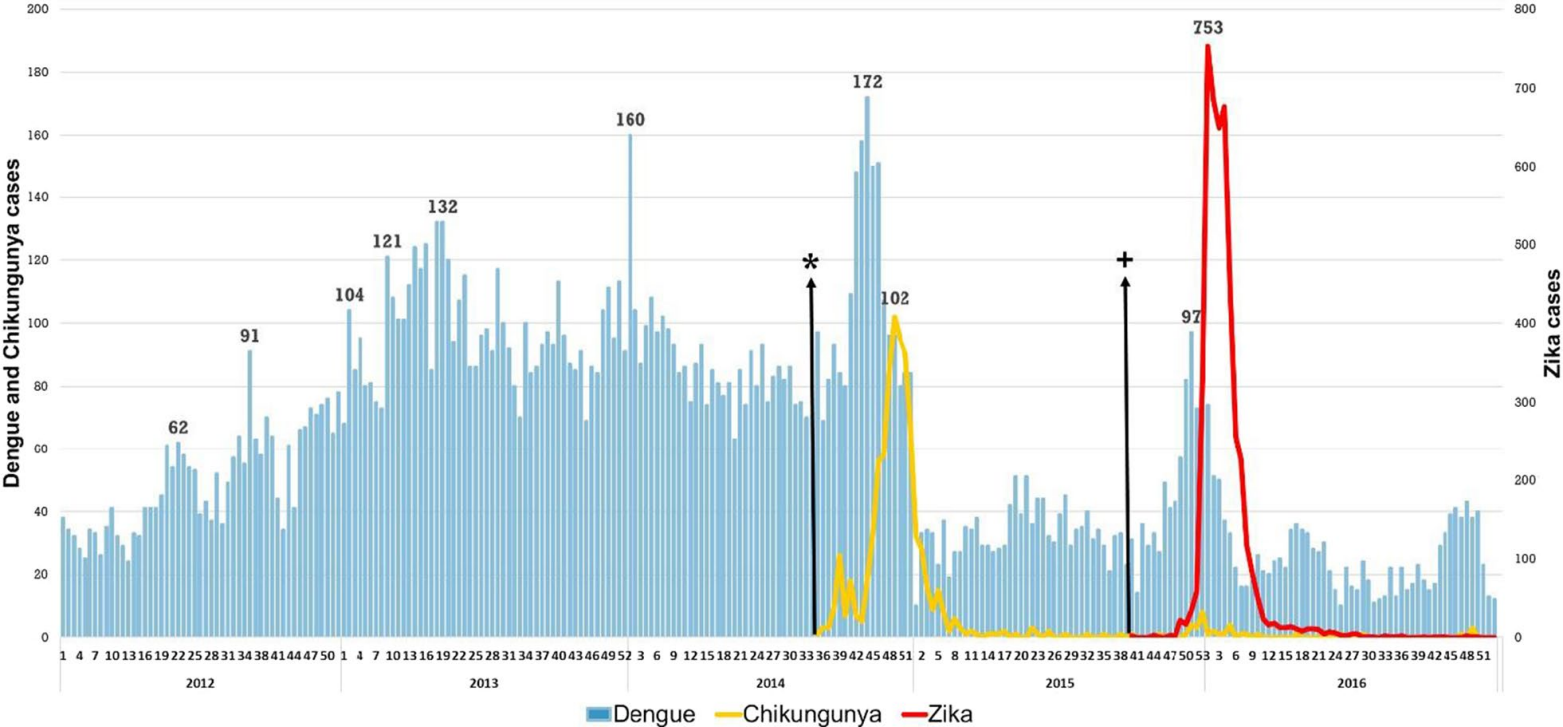
Time series of analytical cases of dengue, chikungunya and Zika in the urban area of Cúcuta, Columbia during the period 2012–2016*. Dengue is in blue; chikungunya is in yellow and Zika is in red. The cases of Zika are quantified on the right. *Week of the first case of chikungunya; + Week of the fist case of Zika

**Fig. 5:**
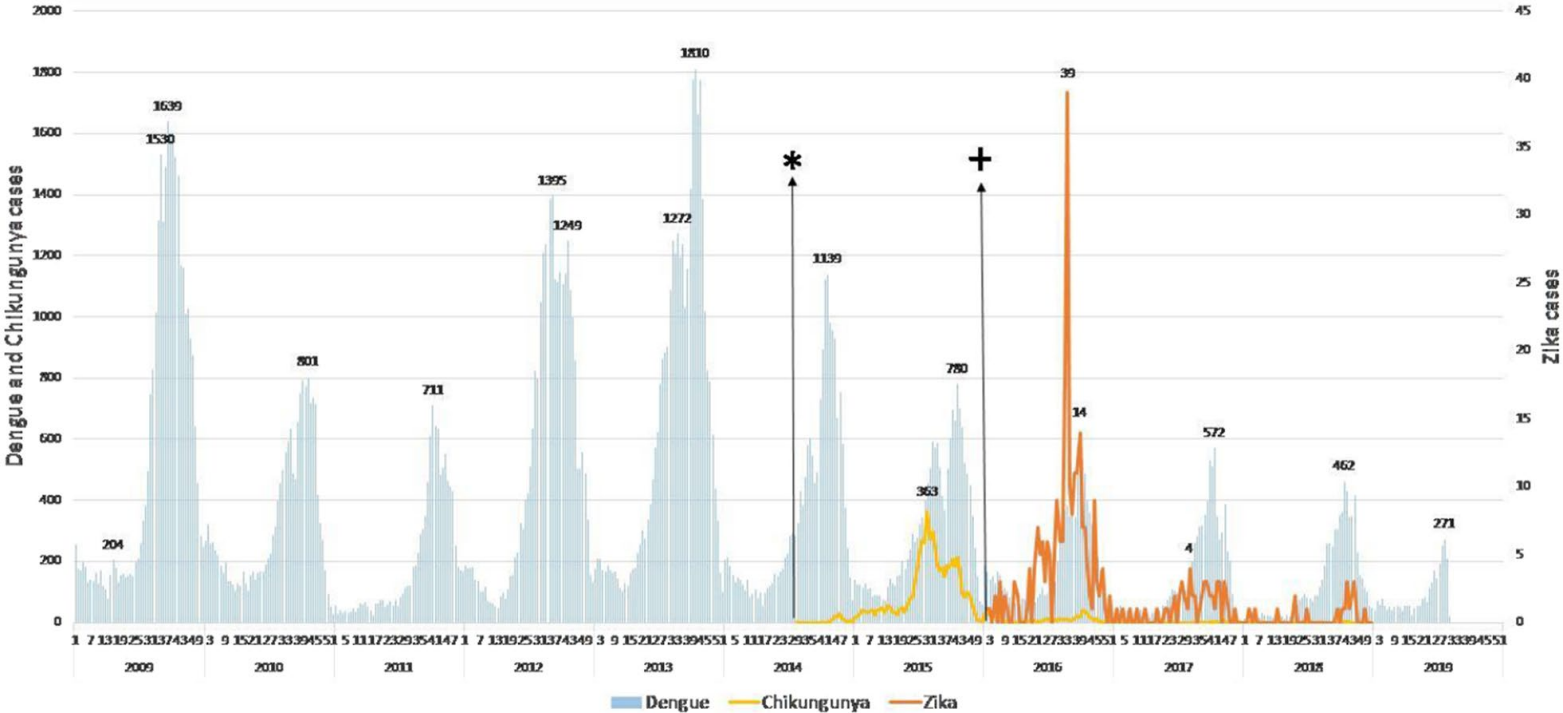
Time series of cases reported weekly in Mexico, of cases of dengue, chikungunya and Zika during the period 2009–2019. Dengue is in blue; chikungunya is in yellow and Zika is in red. The cases of Zika are quantified on the right. *Week of the first case of chikungunya; + Week of the fist case of Zika

In order to better understand the importance and challenges of outbreak prediction (as done by the EWARS) we will first present an overview of the dengue, chikungunya and Zika outbreaks which occurred during the period of our analysis (Figs. 4 and 5).

#### Dengue

For Colombia, 15,811 cases of dengue were included in the analysis, 2013 was an epidemic year with 820 cases per 100,000 population in Cúcuta city, followed by the 2014 outbreak with 785 cases per 100,000 inhabitants. Another increase in dengue was observed before the onset of the 2015 Zika outbreak, however it is likely that in the weeks leading up to the confirmation of the first Zika case in the city, many suspected dengue cases would have actually been Zika. In Mexico, 2012 and 2013 were epidemic years for dengue with 1060 cases/100,000 inhabitants and 1300 cases/100,000 inhabitants confirmed cases respectively. After those epidemic years Mexico has seen a continuous reduction of dengue cases until 2018 with 12,706 confirmed cases.

#### Chikungunya

During the chikungunya outbreak in 2014–2015, cases were recorded from week 35 of 2014. An epidemiological peak was observed for week 48 and a downward curve with stabilization of transmission from week 10 of 2015. For the present study, 863 chikungunya cases with individual records required by EWARS were included. From the data considered for this study, the incidence rates of 115 cases/100,000 population in 2014 and 20 cases/100,000 population in 2015 were estimated. 128 cases were recorded in 2016. In Mexico, although the first cases appeared during 2014 (222 in the whole country) the chikungunya outbreak occurred during 2015 with 12,588 confirmed cases. After 2015 there has been a steady reduction with 759 confirmed cases in 2016 to only 39 confirmed cases in 2018. The peak of the 2015 chikungunya outbreak occurred around week 29. Unfortunately, in Mexico the weekly numbers of chikungunya cases were not available so that the analysis with the EWARS tool could not be conducted (Figs. 4, 5).

#### Zika

The Zika outbreak in Cúcuta started in week 51 of 2015 and lasted until week 10 in 2016 (12 weeks). The peak of cases occurred in week one of 2016 (753 cases), and a total of 4605 cases were reported during the years 2015 and 2016. The highest incidence rate was 69,114 cases per 100,000 population. In Mexico the Zika outbreak, after some sporadic cases in 2015, occurred during 2016 with 7560 confirmed cases in the whole country. 2017 also presented a Zika outbreak which was considerably smaller (3260 confirmed cases). During 2018 the negative trend continued and there were only 860 confirmed cases [15] (Figs. 4, 5).

In the 2013 dengue outbreak, the highest incidence rates in Cúcuta were 81,994 cases per 100,000 population but when chikungunya entered, dengue rates decreased to 21,519 cases per 100,000 population. Similarly, chikungunya rates were lowest, when the Zika outbreak occurred with 69,114 cases per 100,000 population. This phenomenon is worth to be investigated further.

#### Findings from the EWARS application

The datasets of all three arbovirus disease outbreaks were processed using the EWARS tool. The summary of the model calibrations, parameters including the sensitivity, PPV and lag weeks (i.e. period from alarm to start of outbreak) for each disease per country are presented in Table 1. The number of ‘outbreaks’ and of ‘alarms’—which are the basis for calculating the sensitivity and PPV values—are displayed in the tables as well as the alarm thresholds to show their variation for the three diseases.

**Table 1:** Sensitivity and PPV for dengue outbreak prediction using hospitalized cases as outbreak indicator; for chikungunya outbreak prediction using probable cases as outbreak indicator; for Zika outbreak prediction, using defined and probable cases as outbreak indicators

Dengue outbreak							
Country	Alarm indicators	Sensitivity (%)	PPV (%)	No. of outbreaks	No. of Alarms	Alarm threshold	Lag week
Colombia	Mean temp	86	61	76	106	0.69	3
	Rainfall	74	51	76	110	0.70	3
	Humidity	80	60	76	102	0.65	3
	Probable cases	91	50	77	141	0.75	5
	Multiple indicators*	92	68	50	68	0.70	3
Mexico**	Mean temp	81	72	–	–	–	–
	Rainfall	87	65	–	–	–	–
	Humidity	94	50	–	–	–	–
	Probable cases	100	83	–	–	–	–
	Multiple indicators*	84	77	–	–	–	–
Chikungunya outbreak							
Colombia	Mean temp	77	71	13	14	0.80	10
	Rainfall	93	48	14	27	0.45	12
	Humidity	92	85	15	13	0.75	12
	Multiple indicators*	92	92	12	12	0.74	13
Zika outbreak							
Colombia	Mean temp	100	11	2	19	0.05	10
	Rainfall	50	54	2	7	0.05	6
	Humidity	50	11	2	9	0.06	10
	Multiple indicators*	100	14	2	14	0.05	10
Mexico	Mean temp	92	100	36	33	0.50	4
	Rainfall	97	94	28	28	0.40	4
	Humidity (daylight)	88	86	53	52	0.50	5
	Humidity (night)	97	98	29	29	0.50	5
	Positive ovitrap	92	97	25	25	0.40	5
	Average Egg counts	78	77	22	22	0.40	4

*Multiple indicators; temperature, precipitation and humidity, *PPV* positive predictive value

**Values from Mexico taken from a previous period [14]

#### Dengue

A value of z = 1.0 was found in the Colombian and Mexican data sets to be the most suitable (i.e. producing the highest sensitivities and PPV values) multiplier of the SD to define the upper limit of the endemic channel with “hospitalized dengue cases” as outbreak indicator. The sensitivity to correctly predict a dengue outbreak varied between 74 and 92% in Colombia and 81–100% in Mexico, for different alarm indicators; whereas the PPV ranged between 50 and 68% in Colombia and 50–83% in Mexico, including both single and multiple alarm prediction analysis. The Table 1 shows that multiple alarm predictors enhanced the prediction model. The lag time in Colombia ranged from 3 to 5 weeks ahead of an outbreak. Table 1 provides detailed results. The calculations for dengue in Mexico (taken from a previous analysis) were done in exactly the same way as the calculations in Colombia. Unfortunately, we did not get a new data set from Mexico to be able to repeat the dengue analysis.

#### Chikungunya

Due to insufficient numbers of hospitalized cases found in the Colombian surveillance dataset (as most cases were mild), ‘probable cases’ were used as the outbreak indicator instead, which revealed a sensitivity range of 77–93% and a PPV range of 48–92%. The lag time between positive alarm and start of the outbreak was 10 to 13 weeks, much longer than observed with dengue (Table 1).

#### Zika

Estimates of both sensitivity and PPV for different alarm indicators ranged in Colombia from 50 to 100% (mean temperature and multiple indicators with the highest sensitivities) and from 11 to 54% respectively (rainfall with the highest PPV). In Mexico, the range was from 78 to 97% (rainfall with the highest sensitivity) and 77–100% (mean temperature with the highest PPV) respectively (Table 1). In general, the alarm coefficients were stronger in Mexico compared to those in Colombia. The historically higher number of outbreaks and alarm thresholds in Mexico compared to Colombia were reflected in improved sensitivity and PPV values for predicting Zika outbreaks. The difference may also be caused by differences in data quality which was higher in Mexico. The lag times were longer in Colombia—ranging from 6 to 10 weeks—compared to Mexico—ranging from 4 to 5 weeks.

## Discussion

### Outbreak pattern

The observation of the outbreaks of dengue, chikungunya and Zika in Colombia and Mexico suggests that arboviruses present a similar pattern at the start of the outbreak. The increase of the chikungunya and Zika curves was preceded by a dengue peak, possibly due to diagnostic confusion (see Figs. 4, 5). It can be assumed that during the first weeks of the chikungunya outbreak in Colombia, the unusual increase of dengue cases (172 cases in week 44 of 2014) was possibly due to chikungunya infections that remained unconfirmed by the laboratory. The later chikungunya cases were correctly diagnosed and the epidemic curve rapidly increased.

The same happened when the Zika outbreak occurred. Two weeks before the peak of Zika cases, there was an increase in “dengue cases”. Many of these were probably Zika infections. Only after the laboratory confirmation of the Zika virus in the national laboratory, the correct diagnoses were notified and the number of reported Zika cases increased. A similar but less pronounced phenomenon was observed in Mexico in 2014/15: at the beginning of the chikungunya outbreak, there was an increased dengue activity probably because the medical doctors did not consider chikungunya to be a diagnostic option. The same happened with the Zika outbreak in 2016 when first dengue case numbers increased and subsequently Zika was detected. The delay in the confirmation of cases can lead to the silent spread of the new disease, which makes control difficult. This situation highlights the need for an improved surveillance system with a focus on early outbreak warning.

The observation that *Aedes* borne arbovirus outbreaks seem to mutually suppress each other warrants further confirmation.

Dengue, chikungunya and Zika have a seasonal transmission pattern which is accounted for in the present EWARS model. However, there are also important variations over the years depending on response activities by the vector control services and climatic change. These variables are now taken care of in a further developed EWARS model, which is being tested at the moment and will be published later.

### The propose of Early Warning and Response System (EWARS)

The EWARS tool is primarily aimed at supporting district health managers and national health planners to mitigate or prevent disease outbreaks, ideally using tools that are integrated in the national surveillance programs [26]. To further ensure effective functions, the EWARS should be perceived as an information system designed to support the decision-making of national- and local-level institutions but also enable vulnerable groups in the society to take actions to mitigate the impacts of an impending risk [26].

Recent analysis of the evidence indicates that early warning and response system that are capable of demonstrating evidence of prospective predictive ability and allows technical and practical adaptations of local public health responses while augmenting communications channels between users at central and district levels are tools that are more likely to be implemented into national surveillance programs [27]. In this sense, EWARS has moved towards frameworks that facilitate low-cost IT maintenance and adapt to unskilled users. The aim is to form a tool that can be plausibly integrated into existing national systems [25, 27].

### Predictive abilities of the Early Warning and Response System

In an effort to more effectively prepare for and prevent arbovirus disease outbreaks, the EWARS appeared to adequately predict outbreaks of dengue (3 to 5 weeks), chikungunya (10 to 13 weeks) and Zika (6 to 10 weeks) ahead of time in Colombia. In Mexico, the lag time was relatively shorter for Zika with 4 to 5 weeks ahead of the outbreak (not tested for dengue and chikungunya). The variation of lag times for the three diseases may be due to different extrinsic incubation periods (i.e. the time the virus replication and passage from the mosquito gut to the salivary gland requires) but could also be due to the different delays caused by health services diagnose of the diseases [10]. A lag time of 6 to 10 weeks ahead of disease outbreaks would allow timely public health services response with enhanced vector control measures. Nevertheless, shorter time periods for preparing response activities will require the definition of high transmission “hot spots” where interventions can be targeted. The better the control programmes are prepared to identify such high transmission areas and step up the response quickly, the better the chance to mitigate or even avert an outbreak. This has been shown for Mexico [28] and has triggered the incorporation of risk maps into the EWARS tool.

For dengue outbreaks, the sensitivity of alarm indicators to correctly predict an outbreak varied in both countries, with a validity ranging between 74 and 92% using multiple meteorological indicators. The importance of meteorological alarm indicators underlines the characteristic climate sensitivity of vector borne diseases. This pattern is similar to that observed in other studies, which used EWARS for dengue outbreak prediction (83–99% sensitivity in Brazil, 50–99% in Malaysia and 79–100% in Mexico) [14]. Likewise, the PPV of up to 68% in Colombia and up to 83% in Mexico was similar to those reported in previous reports (40–88% in Brazil, 71–80% in Malaysia and 50–83% in Mexico) [14]. The sensitivity and PPV as statistical measures of EWARS performance have meaningful operational implications with the sensitivity indicating the validity of the tool in detecting and predicting outbreaks in time. The PPV, however, can inform local health district managers of potential economic consequences of failing positive alarms—e.g. for 70% PPV, one can be certain that about 30% of resources deployed would not be efficiently used. This is a crucial measure which has further directed the EWARS design to employ a step-wise approach of vector control and response to ensure more efficient application of the tool mainly in resource-limited settings (‘initial’ response: is declared when two consecutive alarm signals occur; ‘early’ response: is declared when three consecutive alarm signals occur; and ‘late’ response: is declared when more than three consecutive outbreak weeks take place). Nevertheless, even in the case of false alarms, resources on vector control are not spent in vain as they contribute in any case to keeping vector densities down.

In the case of chikungunya, the sensitivity to correctly predict an outbreak varied among alarm indicators from 77 to 93%, being rainfall and the combination of meteorological indicators the alarm signals with the highest sensitivity. Likewise, the PPV of up to 85% was similar to those reported with dengue in other countries [14]. Therefore, the EWARS would predict outbreaks 10 to 13 weeks in advance, providing adequate time to activate response actions. In the case of Zika, the sensitivity (50–100%) and PPV (11–100%) were similar to the prediction of dengue and chikungunya when using alarm indicators with the highest values for sensitivity and PPV. While several studies are existing in the literature to demonstrate the applications of prediction models for dengue outbreaks, the EWARS is also useful for Zika and chikungunya outbreak prediction. Based on a recent scoping review study [27], five models showed outstanding performance in dengue outbreak prediction; (i) the dynamic risk maps absolute shrinkage and selection operator (LASSO) processing multiple meteorological information; (ii) the auto-regression integrated moving average (ARIMA) using meteorological information and Google Trends data; (iii) the Shewhart moving average regression model (SMAR) maintaining a combination of meteorological, epidemiological, and entomological alarm indicators, which is the model employed in our study; (iv) the seasonal autoregressive integrated moving average (SARIMA); and (v) the stochastic Bayesian maximum entropy (BME) model using a mix of meteorological and entomological alarm indicator. Despite the fact that LASSO models seem to be toping the tools performance, these models were declared unamenable to easy and direct interpretation and usually demand advance-level of historical data, which limits their applications. No studies were retrieved from the literature to support the statistical prediction performance of Zika and chikungunya outbreaks.

This is the first study to evaluate TDR-EWARS using chikungunya and Zika surveillance data against meteorological and entomological alarm information, and the overall study findings were promising towards implementing the tool at national level. However, outbreak prediction in low endemic areas is less optimal and should be carefully interpreted in relation to routine vector control and response. Despite the fact that 3-year data records were sufficient to demonstrate the applicability of EWARS to broader *Aedes* borne arbovirus diseases, using longer historical surveillance records has the potential to affect the definition of outbreaks and consequently impact on the sensitivity and PPV. As the user-friendliness of the application has already been established [14], the next step is to bring it to practical use in endemic countries and monitor its ability to predict outbreaks and trigger effective response.

### Limitations of the study

This study was based on the cases registered by the public health surveillance system in Colombia, which classifies the cases as probable, confirmed and hospitalized [1, 19–21]. All confirmed cases should present a positive laboratory test, but in the current Colombian database the confirmed cases were sometimes based on clinical criteria. For chikungunya in Colombia, 106,592 cases were reported by SIVIGILA in 2014, 98% of which were confirmed cases according to clinical criteria [19–21]. This reduces the reliability of the case definition. For dengue, the use of hospitalized cases as outbreak indicator was feasible, but in chikungunya and Zika this was hardly the case due to the low proportion of “hospitalized cases”. This limitation was overcome by taking into account probable and confirmed cases as outbreak indicators a suggested by other authors [14, 19–21, 23, 24]. Furthermore, there is a possibility of overestimation caused by the out-of-sample prediction, which may explains the case of 100% sensitivity observed. This can be reduced by employing a longer-data history of disease and alarm information.

In Cúcuta, more than 23,000 cases of chikungunya occurred, but the majority were diagnosed collectively (“collective reporting” i.e. all patients with fever and other symptoms in the waiting area of a health service are diagnosed as having chikungunya), and only a small proportion of cases had complete information from individualized examination [19, 22] which is the routine approach in Mexico. Collective reporting demonstrates the impact of the chikungunya outbreak on the overstretched health system in Colombia. However, the data analyzed in this study was collected throughout the epidemic underlining the ability of the surveillance system to function under difficult circumstances [19, 29].

The time period for retrospectively testing the validity of the EWARS tool was relatively short (3 years in the case of Zika); longer retrospective observation periods would have reflected better the usual pattern of the disease which is not possible in case of a newly emerging disease.

## Conclusion

EWARS demonstrated promising capability of timely disease outbreak prediction with an operational design likely to improve the coordination among stakeholders. However, the prediction validity varied substantially across different types of diseases and appeared less optimal in low endemic settings.

## Data Availability

The epidemiological data that support the findings of this study are available from the Arbovirosis Surveillance Systems in Colombia and Mexico, but restrictions apply to the availability of these data, which were used under license for the current study, and so are not publicly available. Data analysed during the current study are available from the corresponding author (RC) on reasonable request and with permission of CENAPRECE, Mexico and IDS-Norte de Santander, Colombia.

## Abbreviations

CHIKV: Chikungunya virus
EWARS: Early Warning and Response System.
INS: National Institute of Health (for Instituto Nacional de Salud)
MoH: Ministry of Health
SD: Standard deviation
SINAVE: National Epidemiological Surveillance System (for “Sistema Nacional de Vigilancia Epidemiológica”)
SIVIGILA: National Public Health Surveillance System (for “Sistema Nacional de Vigilancia en Salud Pública”)
PPV: Positive predictive value
TDR-WHO: The Special Program for Research and Training in Tropical Diseases
WHO: World Health Organization
ZIKV: Zika virus
